# Estradiol Protects Female ApoE KO Mice against Western-Diet-Induced Non-Alcoholic Steatohepatitis

**DOI:** 10.3390/ijms24129845

**Published:** 2023-06-07

**Authors:** Layanne C. C. Araujo, Alessandra G. Cruz, Felipe N. Camargo, Felipe G. Sucupira, Gabriela V. Moreira, Sandro L. Matos, Andressa G. Amaral, Gilson Masahiro Murata, Carla R. O. Carvalho, Joao Paulo Camporez

**Affiliations:** 1 Department of Physiology, Ribeirao Preto School of Medicine, University of Sao Paulo, Ribeirao Preto 14049-900, Brazil; 2 Department of Physiology and Biophysics, Institute of Biomedical Sciences, University of Sao Paulo, Sao Paulo 05508-000, Brazil; 3 Department of Medicine, School of Medicine, University of Sao Paulo, Sao Paulo 01246-903, Brazil

**Keywords:** estradiol, NASH, Western diet, ovariectomy, female ApoE KO mice

## Abstract

The prevalence of non-alcoholic fatty liver disease (NAFLD) and its severe form, non-alcoholic steatohepatitis (NASH), is higher in men than in women of reproductive age, and postmenopausal women are especially susceptible to developing the disease. Aim: we evaluated if female apolipoprotein E (ApoE) KO mice were protected against Western-diet (WD)-induced NASH. Methods: Female ovariectomized (OVX) ApoE KO mice or sham-operated (SHAM) mice were fed either a WD or a regular chow (RC) for 7 weeks. Additionally, OVX mice fed a WD were treated with either estradiol (OVX + E2) or vehicle (OVX). Results: Whole-body fat, plasma glucose, and plasma insulin were increased and associated with increased glucose intolerance in OVX mice fed a WD (OVX + WD). Plasma and hepatic triglycerides, alanine aminotransferase (ALT), and aspartate aminotransferase (AST) hepatic enzymes were also increased in the plasma of OVX + WD group, which was associated with hepatic fibrosis and inflammation. Estradiol replacement in OVX mice reduced body weight, body fat, glycemia, and plasma insulin associated with reduced glucose intolerance. Treatment also reduced hepatic triglycerides, ALT, AST, hepatic fibrosis, and inflammation in OVX mice. Conclusions: These data support the hypothesis that estradiol protects OVX ApoE KO mice from NASH and glucose intolerance.

## 1. Introduction

Non-alcoholic fatty liver disease (NAFLD) has been characterized as a public health problem, affecting approximately 6–45% of the general population. This percentage increases to 70% when individuals have type 2 diabetes and 90% when morbidly obese. NAFLD can be classified according to histological parameters into non-alcoholic steatosis, characterized by the accumulation of lipids in hepatocytes, and non-alcoholic steatohepatitis (NASH), which displays steatosis, cellular ballooning, inflammation, and hepatic fibrosis. Moreover, NASH can progress to cirrhosis and hepatocellular carcinoma [1,2].

In the last decades, data from clinical studies as well as experimental studies revealed that, in addition to its enormous role in sexual development and reproduction, estradiol contributes enormously to glycemic homeostasis [3,4,5]. Reduced estrogen concentration during menopause is associated with increased visceral fat and, in turn, metabolic diseases such as insulin resistance, type 2 diabetes, and cardiovascular disease. Additionally, insulin sensitivity is higher in women before menopause than in men [6], and estrogen replacement therapy in postmenopausal women reduces the risk of developing type 2 diabetes [7]. NAFLD has also been associated with the period of menopause, showing that reduced levels of estradiol in postmenopausal women were associated with NASH development, regardless of age and waist circumference. This relationship has also been shown in animal models, such as ovariectomized (OVX) female rodents fed a high-fat, high-cholesterolemic diet [8,9].

Experimental studies also demonstrate the importance of estradiol in metabolic homeostasis. An increase in body weight associated with increased body fat is observed in both OVX female rats [10] and female mice [3]. Furthermore, male and female mice lacking the aromatase enzyme showed increased body weight, body fat, and adipocyte hypertrophy [11]. In fact, we demonstrated that estradiol treatment in OVX female mice fed a high-fat diet reduced body weight and fat percentage and increased insulin sensitivity [3]. This effect is also observed in high-fat-fed male mice treated with E2 [4].

Apolipoprotein E (ApoE) is responsible for the transport of lipids between various cells and tissues in the body, being a key regulator of plasma lipid levels and participating in the homeostatic control of lipid content in the plasma and tissue. One study reported that ApoE KO mice displayed increased inflammation and cholesterol levels compared with wild-type mice. Additionally, ApoE KO mice fed a high-fat diet (HFD) or Western diet (WD) were more prone to the development of metabolic syndrome and NASH [12,13,14,15,16]. Therefore, male ApoE KO mice fed a WD for seven weeks seem to be a suitable experimental model of NASH associated with obesity and insulin resistance [16,17], reproducing the human NASH pathology.

Taken together, this study aims to evaluate the potential protective effect of estradiol against the development of NASH in a rodent model. We hypothesize that OVX mice with reduced estradiol concentration would be prone to the development of NASH when fed a WD. In contrast, estradiol replacement would protect OVX mice against WD-induced NASH.

## 2. Results

### 2.1. OVX ApoE KO Mice Are Prone to Western-Diet-Induced Non-Alcoholic Steatohepatitis

**Figure 1:**
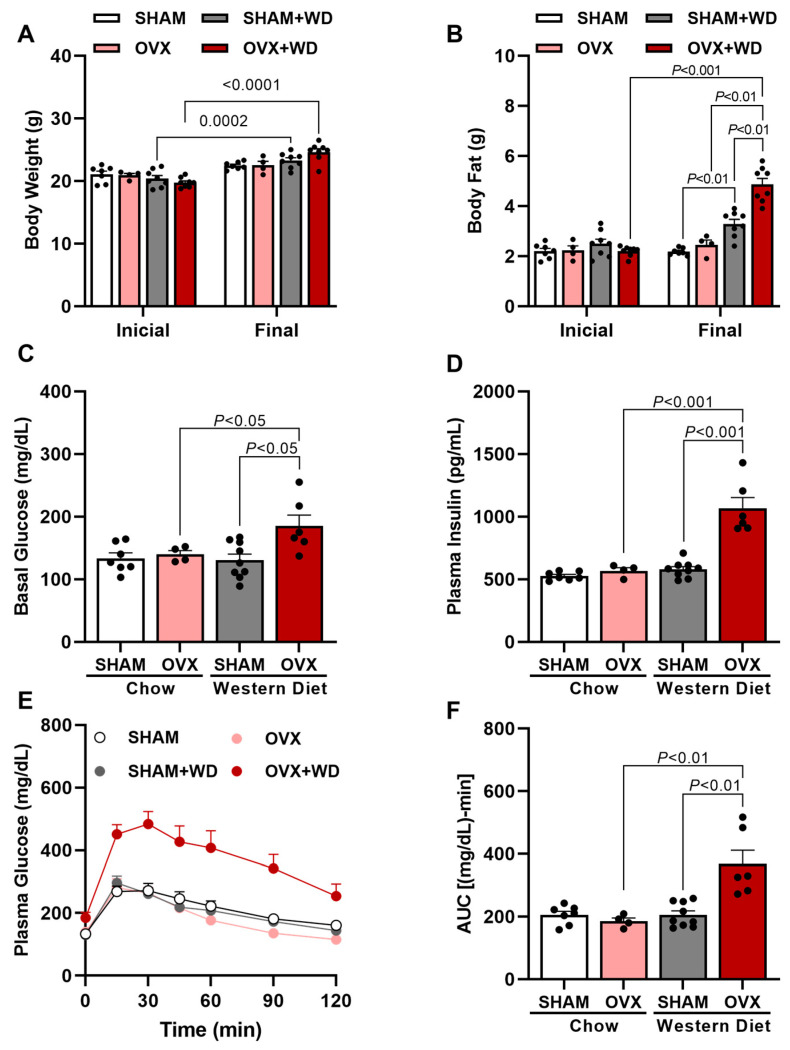
Western-diet-fed ovariectomized mice displayed increased body fat, basal glucose, plasma insulin, and glucose intolerance. Initial body weight (before surgery and diet) and final body weight (after 7 weeks of WD) of SHAM mice, SHAM mice fed with Western diet (WD) (SHAM + WD) for 7 weeks, OVX mice, and OVX mice fed with Western diet (WD) for 7 weeks (OVX + WD) (**A**). Initial body fat (before surgery and diet) and final body fat (after 7 weeks of WD) (**B**). Basal glucose (**C**). Plasma insulin (**D**). Plasma glucose (**E**). Area under curve (AUC) of plasma glucose in different times (**F**). Data are represented as mean ± SEM. The statistical differences as indicated by two-way ANOVA.

Before surgery and the WD intervention, the groups had no differences in body weight and fat mass (Figure 1A,B). After ovariectomy, OVX mice fed a WD displayed increased body weight gain associated with higher whole-body fat compared with both WD-fed SHAM mice and RC-fed OVX mice (Figure 1A,B), showing that estrogen deprivation in OVX mice leads to obesity when fed a diet rich in fat and cholesterol. Associated with the increased body weight and fat, OVX mice displayed higher fasting glucose and insulin after seven weeks of WD feeding compared to WD-fed SHAM and RC-fed OVX mice (Figure 1C,D). Corroborating with these data, OVX mice also showed elevated glucose intolerance compared with WD-fed SHAM mice and RC-fed OVX mice observed during a glucose tolerance test (Figure 1E,F).

**Figure 2:**
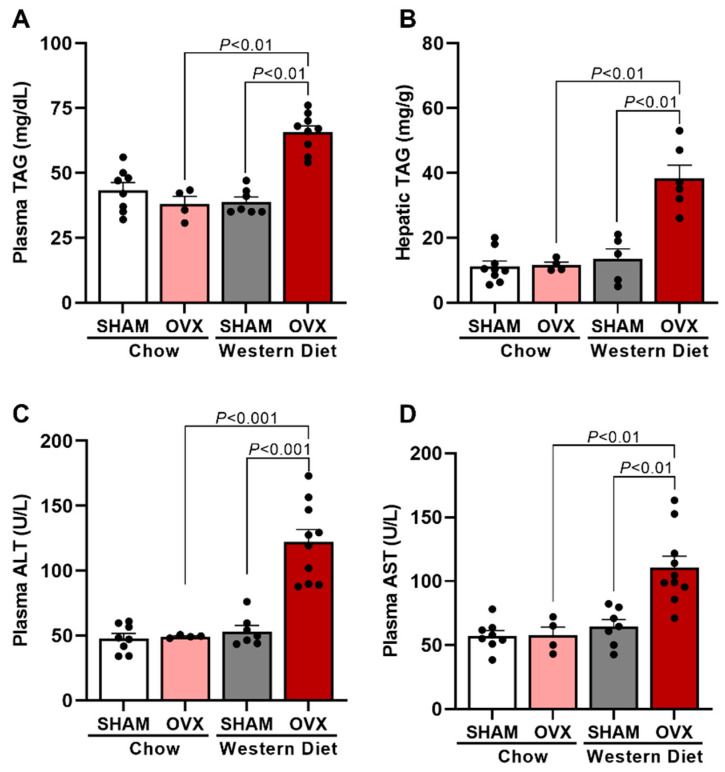
Western-diet-fed ovariectomized mice displayed increased liver triglycerides and hepatic enzymes and triglycerides in plasma. Plasma triglycerides (TAG) (**A**). Hepatic TAG (**B**). Plasma alanine aminotransferase (ALT) (**C**). Plasma aspartate aminotransferase (AST) (**D**). Data are represented as mean ± SEM. The statistical differences were indicated by two-way ANOVA.

Usually, obesity and insulin resistance, as displayed by WD-fed OVX mice, are associated with dyslipidemia and ectopic lipid deposition [18,19]. Therefore, both plasma TAG and hepatic TAG content in these mice was measured. OVX + WD mice displayed higher plasma TAG concentration (Figure 2A) and higher hepatic TAG content (Figure 2B) than WD-fed SHAM mice and RC-fed OVX mice. Interestingly, it was also observed that OVX-WD mice showed higher plasma concentration of hepatic enzymes, ALT and AST, compared with SHAM + WD and OVX groups (Figure 2C,D), suggesting that these mice, with estrogen deprivation, have liver damage after 7 weeks of WD feeding.

**Figure 3:**
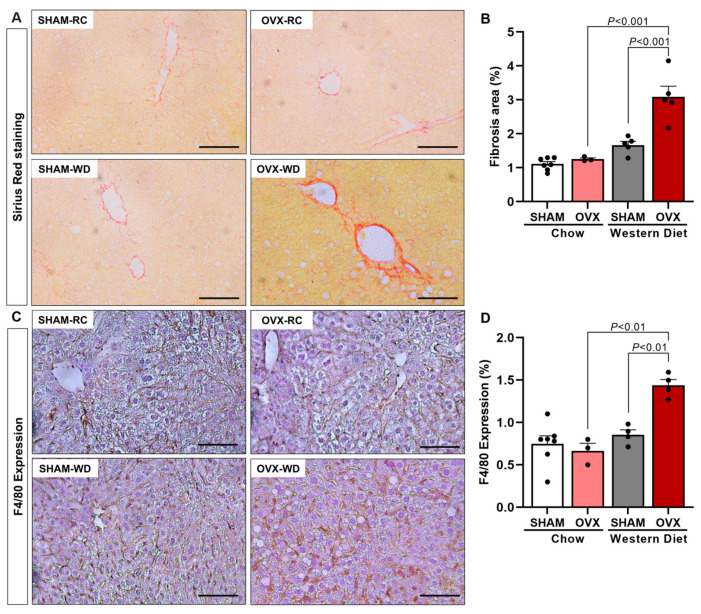
Western-diet-fed ovariectomized mice displayed increased hepatic fibrosis and inflammation. Representative images of picrosirius staining, 20× objective (**A**). Quantification of fibrosis area of red staining, which represents collagen (**B**). Representative images of F4/80 expression, 20× objective (**C**). Quantification of hepatic inflammation represented for F4/80 expression (macrophage activity marker) (**D**). Black bar represents a scale of 100 μm, 20× objective. Data are represented as mean ± SEM. The statistical differences were indicated by two-way ANOVA.

Obesity with insulin resistance and glucose intolerance associated with dyslipidemia and hepatic steatosis, displayed by OVX + WD mice, translates to what is usually observed in humans with NAFLD [1,20]. Some NAFLD patients (~20%) may develop a more severe stage of this disease, which is NASH, also displaying liver fibrosis and inflammation [20,21]. Since male ApoE KO mice fed a WD is a rodent model of NASH [16,17], these parameters were also measured in our study. OVX mice fed a WD significantly increased liver fibrosis and inflammation (Figure 3) compared with SHAM + WD and OVX + RC groups. Interestingly, SHAM mice fed a WD were protected by the original physiological function of the ovary against developing liver fibrosis and inflammation (Figure 3).

### 2.2. Estradiol Protects OVX ApoE KO Mice against WD-Induced NASH

OVX mice fed a WD for 7 weeks displayed all the characteristics of obesity associated with NASH. In contrast, SHAM mice fed the same diet were protected entirely, suggesting that the estrogen deprivation in OVX mice might make these animals prone to WD-induced NASH.

**Figure 4:**
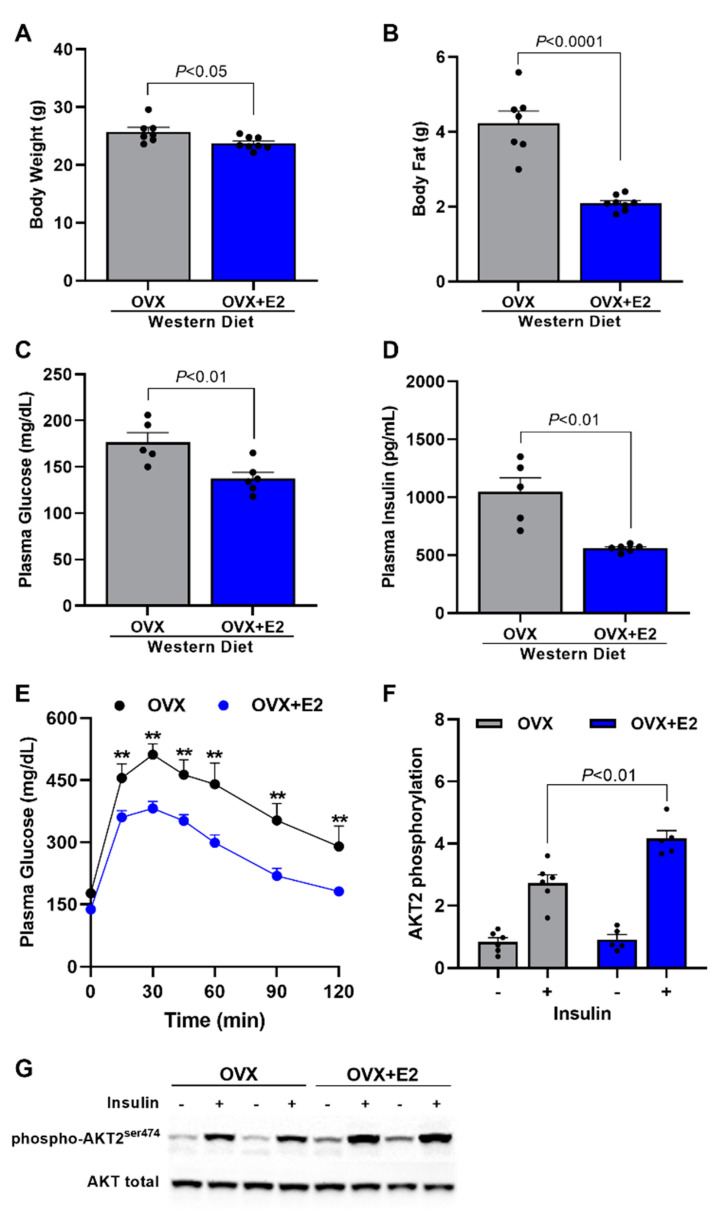
Estradiol reduces body weight and fat, basal glucose, plasma insulin, and glucose intolerance. Body weight (**A**). Body fat (**B**). Fasting glucose (**C**). Fasting plasma insulin (**D**). Plasma glucose during a glucose tolerance test (GTT) (**E**). Quantitative analysis of hepatic AKT2 phosphorylation (**F**). Western blot representative images of hepatic AKT2 phosphorylation (**G**). Data are represented as mean ± SEM. The statistical differences as indicated by T test (**A**–**D**) or two-way ANOVA (**E**) were as follows: ** *p* < 0.01 represents the difference between OVX vs. OVX + E2.

Therefore, in order to evaluate if it is, in fact, estrogen that protects SHAM mice against WD-induced NASH, we treated OVX mice fed a WD with either vehicle (OVX) or estradiol (OVX + E2). Estradiol-treated mice displayed a slightly reduced, although significant, body weight compared with OVX mice (Figure 4A). They also showed a significant reduction in total body fat of ~50% compared with OVX mice (Figure 4B). Associated with these reductions in body weight and body fat, OVX + E2 mice presented reduced fasting plasma glucose (Figure 4C) and fasting plasma insulin (Figure 4D), suggesting that estradiol leads to a reduction in insulin resistance presented in OVX mice. Corroborating with these data, OVX + E2 mice also displayed increased glucose tolerance (Figure 4E) and increased hepatic insulin-stimulated AKT2 phosphorylation (Figure 4F,G) compared with vehicle-treated mice.

**Figure 5:**
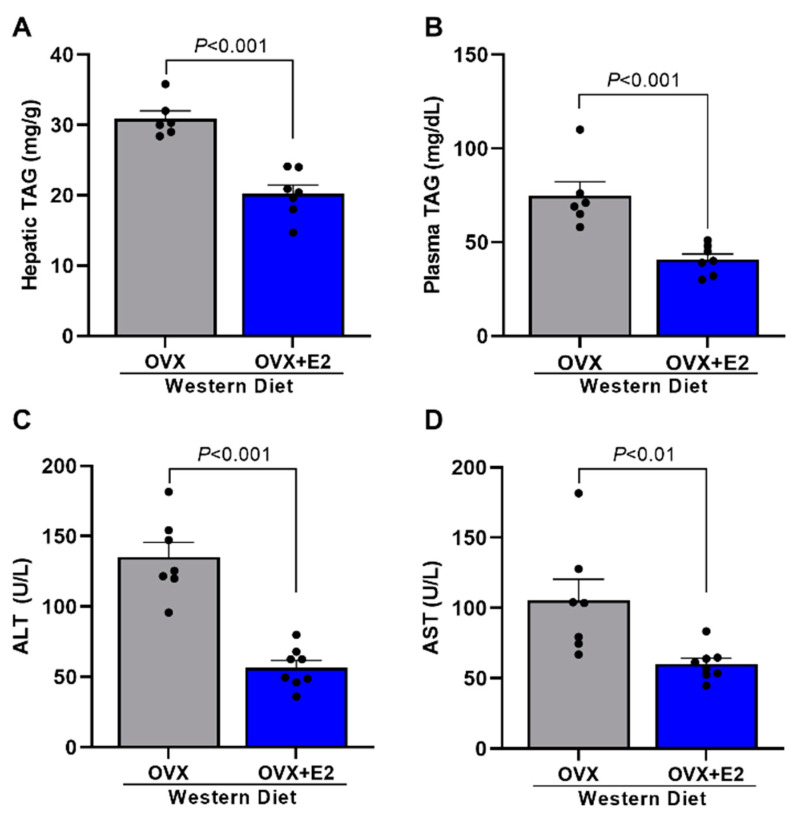
Estradiol reduces liver and plasma triglycerides and hepatic enzymes. Hepatic triglycerides (TAG) (**A**). Plasma TAG (**B**). Plasma alanine aminotransferase (ALT) (**C**). Plasma aspartate aminotransferase (AST) (**D**). Data are represented as mean ± SEM. The statistical differences were indicated by *t* test.

It was also evaluated if estradiol treatment protects mice against the hepatic steatosis and liver damage observed in OVX mice. It is possible to observe that OVX + E2 mice displayed significant reductions in hepatic steatosis (Figure 5A), plasma TAG levels (Figure 5B), and liver damage (Figure 5C,D) compared with OVX mice.

**Figure 6:**
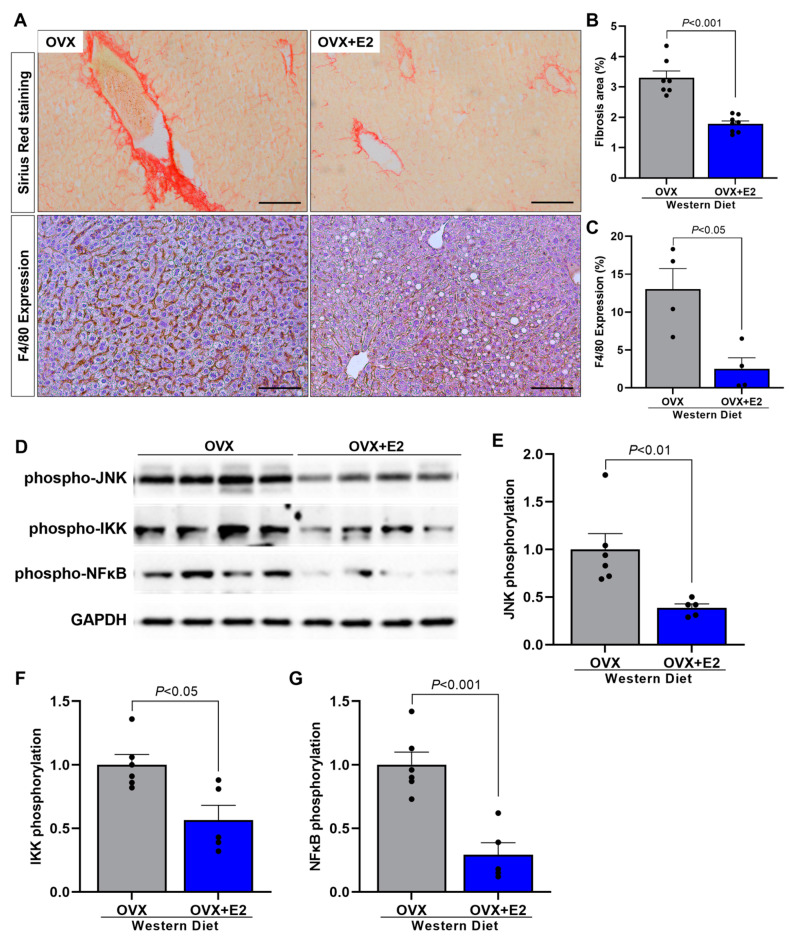
Estradiol reduces hepatic fibrosis and inflammation. Representative images of picrosirius staining, 20× objective, and hepatic inflammation represented for F4/80 expression (macrophage activity marker). Black bar represents a scale of 100 μm, 20× objective (**A**). Quantitative analysis of hepatic fibrosis (**B**). Quantitative analysis of hepatic inflammation represented by F4/80 expression (**C**). Western blot images of hepatic phospho-JNK, phospho-IKK, and phospho-NFκB (**D**). Quantitative analysis of hepatic phospho-JNK (**E**). Quantitative analysis of hepatic phospho-IKK (**F**). Quantitative analysis of hepatic phospho-NFκB (**G**). Data are represented as mean ± SEM. The statistical differences were indicated by *t* test.

In order to evaluate if estradiol treatment also protects mice specifically against NASH characteristics presented in OVX mice, such as fibrosis and inflammation, we also assessed these parameters. OVX + E2 mice displayed significant reductions in hepatic fibrosis (Figure 6A,B) and hepatic inflammation (Figure 6A,C) compared with OVX mice. Corroborating with these data, OVX + E2 mice displayed reduced hepatic JNK, IKK, and NFκB phosphorylation (Figure 6D–G) compared with vehicle-treated OVX mice, suggesting that estradiol has a potential protective effect against WD-induced NASH in female mice.

## 3. Discussion

This study demonstrated that the Western diet associated with ovariectomy promotes increased fat mass, basal glucose, glucose intolerance, and plasma insulin in female ApoE KO mice. In addition to promoting liver damage, OVX mice displayed hepatic inflammation, fibrosis, and increased TAG in the liver and plasma. Estradiol replacement therapy prevented all of this damage caused by diet and ovariectomy in ApoE KO mice.

It has been shown that ApoE KO male mice at 12 months of age have increased serum levels of TAG, glucose, and insulin in addition to hepatic steatosis and increased plasma levels of liver enzymes, demonstrating liver damage [22]. Additionally, 2-month-old ApoE KO male mice fed a high-cholesterol diet (Western diet) showed increases in the same parameters, demonstrating the influence of the diet [23]. It has been shown that 7 weeks of a Western diet in ApoE KO male mice promoted the development of NASH with liver fibrosis [16], and similar effects were observed in our study in OVX ApoE KO female mice. Interestingly, female SHAM mice were protected against Western diet-induced NASH.

Studies have shown that OVX mice fed a high-fat diet (HFD) showed an increase in body weight, fat mass, fasting glycemia and insulin, insulin resistance, and glucose intolerance [3,10], in addition to an increase in plasma TAG [3,24], hepatic steatosis, and fibrosis [25]. Our study showed similar results in OVX ApoE KO mice fed a Western diet, while SHAM mice were protected.

Meng et al. [26] demonstrated in their studies that female ApoE KO mice fed with HFD increased plasma triglycerides and that this increase was even more significant in OVX ApoE KO mice fed with HFD. In addition, the hepatic lipid accumulation was more remarkable in OVX ApoE KO mice when compared with the SHAM group. Hu et al. [27] demonstrated that OVX ApoE KO mice fed with a standard diet also developed an increase in these parameters. These data agree with our results since we demonstrated an increase of TAG in the plasma and liver, confirming hepatic steatosis in OVX ApoE KO mice fed a hypercholesterolemic diet (Western diet). In addition, Jing et al. [28] demonstrated increased body weight of OVX ApoE KO mice fed an HFD, as shown in our study. This increase in body weight in OVX animals observed in our study may be due to the reduction in whole-body energy expenditure, which would lead to a positive energy balance, favoring an increase in body weight associated with increased adiposity and, consequently, an increase in hepatic steatosis. This phenomenon was previously observed in which OVX C57BL6/J mice fed an HFD showed increased body weight, adiposity, and hepatic steatosis [3]. This phenomenon is also observed in animal models, such as animals with a mitochondrial deficiency and, consequently, reduced whole-body energy expenditure associated with increased body weight, adiposity, and ectopic lipid accumulation [19]. These results were associated with an oxidative reduction in the metabolism of white adipose tissue from OVX animals, in which proteins such as UCP-1, Cidea, and PRDM16 (browning markers) in white adipose tissue in OVX animals were reduced, which may explain the decrease in energy expenditure in these mice [3].

Excess lipids can serve as a substrate for the production of lipotoxic species, which can generate reticulum stress and hepatocellular injuries. This lipotoxicity is a significant cause of liver dysfunction and hepatocyte cell death, which stimulates the progression of NASH. Cell death contributes to the development of inflammatory processes and liver fibrosis in the progression of NASH [29]. One study showed that OVX ApoE KO mice fed an HFD increased the pro-inflammatory cytokine (IL-1β) in plasma and lipid deposition in the aorta. This same study reported that IL-1β was increased in postmenopausal women in the smooth muscle and endothelium of the aorta [26]. Liu et al. [30] described increased serum levels of inflammatory factors such as NFKB and AP-1 in OVX ApoE KO mice. Our study showed liver inflammation through macrophage activity, represented by the marker F4/80, hepatic lipid deposition, and hepatic fibrosis in OVX ApoE KO mice with a Western diet.

Our data and several other studies show that ovariectomy has shown essential changes in insulin sensitivity, glucose tolerance, and body weight. Furthermore, when associated with a high-fat diet, it promotes even more damage to these factors and promotes steatosis and liver inflammation. Estrogen plays an essential role in regulating glucose homeostasis, and the deficiency of this hormone is associated with insulin resistance and dysregulation of metabolic homeostasis, contributing to the development of diabetes and obesity [3,4,10,31,32]. Additionally, pieces of evidence have reported that postmenopausal women have an increased risk of diseases such as DM2, cardiovascular disease, and hypertension because their prevalence in postmenopausal women is higher than in premenopausal women [33,34]. Furthermore, women after menopause have an increased risk of glucose intolerance, insulin resistance, hyperlipidemia, and visceral fat accumulation [33,34,35]. E2 receptors are named α and β (ERα, ERβ) and are mainly found in the nucleus. However, ERα mediates most of the metabolic effects of estrogens, which is in a more significant proportion than ERβ in essential tissues for metabolic regulation [36]. Experimentally, ERα-deficient mice showed deleterious responses regarding lipid metabolism, such as decreased fatty acid oxidation and increased de novo lipogenesis, leading to elevated lipid accumulation in the liver compared to control mice [37,38]. These results may be associated with the action of E2 via ERα on mitochondrial dynamics since animals with ERα deletion, specifically in skeletal muscle, showed defects in mitophagy and mitochondrial function in this tissue, leading to reduced O2 consumption [39], suggesting that the lack of ERα action in OVX animals due to the absence of E2 mediates the deleterious results observed in our study.

Studies have shown that hormone replacement in OVX mice with estradiol reduced body weight and blood glucose and improved glucose tolerance and insulin sensitivity [3,40]. These special effects of estradiol are even observed in male mice fed an HFD [4]. Our study observed similar effects with estradiol replacement in OVX ApoE KO mice fed a Western diet. Additionally, it was observed in a study that a reduction of plasma TAG and aortic inflammation through the reduction of NFκB and AP-1 inflammatory pathway proteins in OVX ApoE KO mice fed an HFD when estradiol hormone replacement was applied [30]. Meng et al. [26] also demonstrated in the same animal model an improvement of inflammation in the aorta, reducing serum levels of IL-1β, plasma TAG, and lipid deposition in the aorta, in addition to a reduction of hepatic steatosis with estradiol treatment. Our study also showed reduced plasma and hepatic TAG and liver inflammation and fibrosis in OVX ApoE KO mice fed a Western diet.

In conclusion, these data support the hypothesis that estradiol protects OVX mice from Western-diet-induced NASH and glucose intolerance by protecting female mice against hepatic steatosis, inflammation, and fibrosis.

## 4. Materials and Methods

### 4.1. Animals

Female C57BL/6J apolipoprotein E (ApoE) KO mice, weighing 20–25 g, were obtained from the animal facility at the Biomedical Science Institute of University of São Paulo (ICB-USP) and were maintained in a temperature-controlled room at 22 ± 2 °C with free access to food and tap water and light/dark cycle of 12 h (light on from 6 am to 6 pm). In the first study, mice were divided into four groups: female ApoE KO mice ovariectomized and fed a Western diet (WD) for 7 weeks (OVX + WD), female ApoE KO mice ovariectomized and fed a Regular chow (OVX), female ApoE KO mice sham operated (SHAM) and fed a WD (SHAM + WD), and female ApoE KO mice sham operated and fed a regular chow (SHAM) (Figure 1, Figure 2 and Figure 3). In the second study, mice were divided into two groups: ovariectomized mice fed a WD for 7 weeks and vehicle-treated (OVX), and ovariectomized mice fed a WD for 7 weeks and estradiol-treated (OVX + E2). This second study was designed in order to evaluated specifically the potential protective effect of estradiol (Figure 4, Figure 5 and Figure 6).

The regular chow containing 19%/kcal of protein, 56%/kcal of carbohydrate, 3.5%/kcal of lipids, 5% cellulose, and 4.5% vitamins and minerals, providing 3.2 kcal/kg (PragSoluções Biosciences, Jau, São Paulo, Brazil). The Western diet contained 17%/kcal of protein, 43%/kcal of carbohydrate, and 40%/kcal of lipids, in addition to vitamins and minerals and cholesterol (0.15%), providing 4.6 kcal/kg (D12079B, Reserch Diets, New Brunswick, NJ, USA). It was published that male ApoE KO mice fed a WD for 7 weeks, as performed in our study, is an experimental rodent model of NASH [16,17]. All experimental procedures were performed following the “Guidelines for the ethical use of animals in applied etiology studies” and were previously approved by the Ethics Committee on use of animals at the ICB-USP (CEUA nº 5372180319).

### 4.2. Ovariectomy and Estradiol Treatment

Ovariectomy was performed in ApoE KO female mice at 8 weeks of age as previously published [3]. Female mice were anesthetized with isoflurane (5% induction, 2–3% maintenance). After abdominal incision, the ovaries were clamped and removed. Then the skin was sutured before the animals woke up. In the SHAM group the same procedure was performed, however, the ovaries were not removed.

To study the effect of chronic estradiol administration, OVX mice were sc implanted with pellets releasing either vehicle (OVX) or estradiol (OVX + E2) (0.05 mg for 60 d, i.e., 35 μg/kg per day; Innovative Research of America, Sarasota, Florida) at the same time as ovariectomy, as previously published [3]. During all procedures, mice were in a constant room temperature environment, with 12 h light, 12 h dark cycle, with free access to water and food.

### 4.3. Evaluation of Body Weight and Fat

Total body mass and fat mass were analyzed using the LF50 minispec (Bruker’s, Billerica, MA, USA) in awake mice.

### 4.4. Glucose Tolerance Test

Awake mice were submitted to an intraperitoneal glucose tolerance test (ipGTT) as previously published [41]. After 6 h of fasting, each mouse received an intraperitoneal injection of a solution of 10% glucose (1 mg/g body weight) and the blood samples were collected from a superficial cut in the tail in the following times 0, 15, 30, 45, 60, 90, and 120 min to determine plasma glucose. The blood was applied to reactive strips (Roche^®^, São Paulo, Brazil) and glucose levels were measured using an Accu-chek Active apparatus (Roche, Mannheim, Germany). The plasma glucose at time 0′ was used as fasting plasma glucose. Extra plasma was collected at time 0′ in order to measure basal plasma insulin concentrations by mouse insulin ELISA commercial kit (Mercodia^®^, Uppsala, Sweden). The area under the curve (AUC) was calculating using the GraphPad Prism version 9.0^®^ program (GraphPad Software, La Jolla, CA, USA) considering the individual curve of each mouse.

### 4.5. Measurement of Liver Enzymes in Plasma

After euthanasia, the blood of the animals was collected and centrifuged (15,294× *g*, 2 min) for plasma separation. Then, the plasmatic concentration of liver enzymes (alanine aminotransferase—ALT and aspartate aminotransferase—AST) was analyzed for commercial kit LabTest (Lagoa Santa, Brazil). The dosage of liver enzymes was performed according with the kit protocol.

### 4.6. Tissue Content of Triglycerides

After 6 h of food restriction, the animals were euthanized and the tissues removed for analysis of the lipid content. Tissue triglycerides (TAG) were extracted using the Bligh and Dyer method [42] and measured using a TAG reagent (Bioclin, Belo Horizonte, Brazil).

### 4.7. Liver Histology

#### 4.7.1. Picrosirius Staining

To evaluate liver morphology, as previously published [43], the liver was fixed in 10% formaldehyde solution for 8 h in individual cassettes. Subsequently, the fixed samples were kept overnight in 70% alcohol. The samples were then dehydrated through a series of baths in 95% alcohol, 100% alcohol and xylene. Once the samples were dehydrated, the tissue samples were embedded in paraffin at 60 °C. A microtome (Zeiss, Jena, Germany) was used to cut the samples into 5-micron slices. The slices were stained with picrosirius to identify collagen fibers, and 25 images using 20× magnification (3 distinct slices) from 5 animals per group were analyzed, and 10 images from each animal were obtained using a microscope with 20× objective magnification using a Nikon Eclipse Ti-U microscope coupled with a Nikon DS-R1 digital camera and NIS-Elements BR 3.1 software. The abundance of collagen deposition in the liver was presented as the mean of the percentage from each animal per group. The identification of picrosirius stained area was performed using the ImageJ program. The image was opened in the program, converted to 8-bit grayscale (Image → type → 8-bit). Then the following sequence was performed: Image → Adjust → Threshold; Analyze → Set measurements. The results were obtained through Analyze → Measure [44]. Area values were used as a result, which corresponded to the amount of collagen in the tissue.

#### 4.7.2. Immunohistochemistry (IHC)

For immunohistochemistry studies, as previously published [43], the liver was fixed in a 10% formaldehyde solution for 8 h. After the samples were dehydrated, the tissue samples were embedded in paraffin at 60 °C. Five-micron-thick tissue sections were cut transversely on a microtome (Zeiss, Jena, Germany) and antigen retrieval was performed by heating (96 °C) the specimen sections in sodium citrate buffer. The sections were washed with PB, incubated with 3% hydrogen peroxide to inhibit endogenous peroxide and treated with 10% goat serum for 1 h at 22 °C in a humidified chamber. Next, all the sections were then incubated overnight at 4 °C with the primary rat polyclonal antibody F4-80 (Bio-Rad AbD Serotec, Kidlington, UK) diluted in PBS (1:200). The sections were then incubated with the appropriate secondary antibody (Sigma-Aldrich, St. Louis, MO, USA) for 1 h at RT. For the visualization of immune complexes, the vectastin ABC-kit (Vector Laboratories, Newark, CA, USA) was applied for 1 h at RT for signal amplification and 3,3-diaminobenzidine diluted in PBS containing 0.03% (*v*/*v*) H_2_O_2_ was utilized as the chromogen, yielding the overall brown color. Negative controls were conducted for each antibody by omitting the primary antibody. At least two samples from each animal were independently analyzed by two experienced investigators. Images were acquired with a Nikon DS-R1 digital camera connected to a Nikon Eclipse Ti-U microscope. The immunostained sections were quantified by using the Image Pro Plus V7 software (Media Cybernetics, Rockville, MD, USA). At least 5 areas per slide were selected and photographed in a blinded manner. The expression of F4/80 was calculated as follows: the total area of liver (specimen) was outlined, and the area occupied by cells expressing F4/80 were quantified using the image analyzer within the reference area. A color for F4/80 was pre-defined and applied to the selected area. The result was expressed as a percentage of positive area in relation to the total area. F4/80 is an active macrophage marker, so an increase in the expression of this marker indicates an increase in the inflammatory process.

### 4.8. Immunoblotting

Animals were deeply anesthetized with isoflurane (5%), and after the loss of corneal reflexes, the abdominal wall was opened, and the liver was removed. After that, pieces of the liver (~50 mg) were homogenized in RIPA buffer with protease and phosphatase inhibitor cocktail (Thermo Fisher, Waltham, MA, USA). The tissue extracts were centrifuged at 15,000× *g*, at 4 °C, for 20 min. The protein content of the supernatants was measured by the Bradford method. Aliquots of the supernatant, containing 50 ug total protein, were treated with Novex tris-glycine SDS buffer and RIPA buffer (Thermo Fisher, Waltham, MA, USA), loaded onto Novex WedgeWell 4% and 20% tris-glycine gel (Thermo Fisher, Waltham, MA, USA), and subjected to SDS-PAGE. The proteins were transferred from the gels to nitrocellulose membranes using a Bio-Rad Trans-Blot Semi-Dry (Hercules, CA, USA). The membranes were incubated in TBST-B blocking buffer (10 mM Tris, 150 mM NaCl, 0.05% Tween 20, and 5% skim milk) for 2 h at RT to prevent nonspecific binding to the membrane. The nitrocellulose membranes were incubated with the specific primary antibodies overnight at 4 °C and subsequently incubated with a secondary antibody conjugated to horseradish peroxidase for 1 h at RT. The immunoblots were developed using the SuperSignal^®^ West Pico Chemiluminescent Substrate (BioRad, Hercules, CA, USA). The immunoblots were visualized using an iBright 750 (Thermo Fisher, Waltham, MA, USA), loaded onto Novex WedgeWell 4% and 20% tris-glycine gel (Thermo Fisher, Waltham, MA, USA) and quantified using the ImageJ 1.8.0 software (imagej.net/Downloads). The primary antibodies used were:

- Phosphorylated NFkB (catalog number 3033 S, Cell Signaling, Danvers, MA, USA);
- Phosphorylated Ikkβ (catalog number 2694 S, Cell Signaling);
- Phosphorylated JNK (catalog number 9255 S, Cell Signaling);
- Phosphorylated AKTser474 (catalog number 8599 S, Cell Signaling);
- AKT (catalog number 126811, Abcam, Cambridge, UK);
- GAPDH (catalog number 2118 S).

### 4.9. Statistical Analysis

The results were analyzed using the GraphPad Prism version 9.0^®^ program (GraphPad Software, La Jolla, CA, USA). The minimum sample size per group for each parameter analyzed was defined by a n sufficient to perform the analysis of distribution of samples through the D’Agostino and Pearson omnibus normality test recommended by the GraphPad Prism version 9.0^®^ program (Boston, MA, USA). All samples were evaluated for normal distribution and subjected to either a two-way ANOVA followed by the post hoc Bonferroni multiple comparison test or a Student-*t* test (*p* < 0.05). The results were expressed as mean ± standard error of mean (mean ± SEM).

## Data Availability

The data presented in this study are available on request from the corresponding author.
